# Acetylcholine facilitates localized synaptic potentiation and location specific feature binding

**DOI:** 10.3389/fncir.2023.1239096

**Published:** 2023-11-10

**Authors:** Yihao Yang, Victoria Booth, Michal Zochowski

**Affiliations:** ^1^Department of Physics, University of Michigan, Ann Arbor, MI, United States; ^2^Departments of Mathematics and Anesthesiology, University of Michigan, Ann Arbor, MI, United States; ^3^Department of Physics and Biophysics Program, University of Michigan, Ann Arbor, MI, United States

**Keywords:** acetylcholine, synaptic plasticity, STDP, feature binding, synaptic potentiation

## Abstract

Forebrain acetylcholine (ACh) signaling has been shown to drive attention and learning. Recent experimental evidence of spatially and temporally constrained cholinergic signaling has sparked interest to investigate how it facilitates stimulus-induced learning. We use biophysical excitatory-inhibitory (E-I) multi-module neural network models to show that external stimuli and ACh signaling can mediate spatially constrained synaptic potentiation patterns. The effects of ACh on neural excitability are simulated by varying the conductance of a muscarinic receptor-regulated hyperpolarizing slow K+ current (m-current). Each network module consists of an E-I network with local excitatory connectivity and global inhibitory connectivity. The modules are interconnected with plastic excitatory synaptic connections, that change via a spike-timing-dependent plasticity (STDP) rule. Our results indicate that spatially constrained ACh release influences the information flow represented by network dynamics resulting in selective reorganization of inter-module interactions. Moreover the information flow depends on the level of synchrony in the network. For highly synchronous networks, the more excitable module leads firing in the less excitable one resulting in strengthening of the outgoing connections from the former and weakening of its incoming synapses. For networks with more noisy firing patterns, activity in high ACh regions is prone to induce feedback firing of synchronous volleys and thus strengthening of the incoming synapses to the more excitable region and weakening of outgoing synapses. Overall, these results suggest that spatially and directionally specific plasticity patterns, as are presumed necessary for feature binding, can be mediated by spatially constrained ACh release.

## 1. Introduction

ACh is a neuromodulator that plays an important role in regulating neural excitability and can greatly impact various brain and cognitive functions such as memory consolidation during sleep and attentional control (Marrosu et al., 1995; Parikh and Sarter, 2008; Delorme et al., 2021). Among its varied effects (Picciotto et al., 2012), ACh regulates the excitability of neurons through its action on a muscarine-sensitive M-current (Gu, 2002). This slow, low-threshold $K^{+}$ current can be blocked when ACh is high and results in important modulation of neural response properties such as increasing membrane excitability, altering spike-frequency adaptation, and changing effects on spike timing in response to synaptic inputs (Stiefel et al., 2009; Fink et al., 2013; Roach et al., 2019).

ACh signaling in the neocortex is mediated by projections from the basal forebrain (BF), which, traditionally, has been described as “volume” transmission, namely characterized by relatively low temporal resolution and low spatial heterogeneity (Hasselmo, 1999; Dayan and Yu, 2002; Hasselmo and Giocomo, 2006; Sarter and Lustig, 2019). However, recent anatomical studies indicate that these projections are highly topographically organized (Zaborszky et al., 2005, 2015; Gielow and Zaborszky, 2017; Yuan et al., 2018). In addition, recent amperometric measurements of ACh in the pre-frontal cortex identified spatially localized, transient cholinergic release (Parikh and Sarter, 2006; Sarter et al., 2016) that affected behavioral outcomes in an attentional signal detection task (Howe et al., 2013; Gritton et al., 2016). Thus, in contrast to the traditional view of ACh modulation being a diffusely organized system, this recent experimental evidence demonstrates that ACh signaling can be asynchronous and spatially heterogeneous (Sarter and Lustig, 2019; Disney and Higley, 2020; Yang et al., 2021). Additionally, recent research on imaging of functional gradients of cortical activity during REM sleep in the mouse brain has shown that the spatial distribution of slow waves is determined by regional variation in cholinergic activity (Nazari et al., 2023). Moreover, functional studies have indicated that ACh signaling can be event- or task trial-specific in some neocortical regions as well (Parikh et al., 2007; Sarter and Lustig, 2019).

At the same time, it is known that ACh modulation is critical in cognitive functions like learning and memory storage at both cellular and circuit levels (Hasselmo and Bower, 1993). There is growing evidence that ACh plays a critical role in mediating synaptic plasticity (Seol et al., 2007; Brzosko et al., 2019). Specifically, ACh is an important regulator modulating synaptic plasticity in the hippocampus, the cerebral cortex, and the striatum (Rasmusson, 2000; Partridge et al., 2002). Moreover, an experimental study (Ovsepian et al., 2004) demonstrated that muscarinic receptor activation lowered the threshold for LTP induction.

There is also growing evidence that acetylcholine can mediate feature binding, i.e., the capacity of the brain to selectively link different features of a processed input into one neuronal representation (Botly and De Rosa, 2007, 2008, 2012). Motivated by these results indicating spatial heterogeneity of ACh release and ACh’s role in brain plasticity, here we investigate how spatially constrained ACh signaling affects synaptic reorganization mediated by spike timing dependent plasticity (STDP). We specifically explore the role of spatially heterogeneous cholinergic modulation in facilitating preferential plasticity patterns between distinct network regions, leading to overall network reorganization and a substrate for feature binding. We show that localized regions with high ACh levels relative to synaptically connected network areas can be preferentially potentiated (i.e., the synapses targeting these regions are strengthened) and thus linked together. In this way, the spatial variation in cholinergic activity allows networks to selectively generate potentiation patterns with accuracy and consistency, potentially leading to more efficient learning and memory formation. When ACh levels are uniformly high in connected network regions, reciprocal potentiation of synaptic weights can occur which may correspond to precise, one-to-one information binding between distinct cognitive representations. This effect has the potential to explain the prevalence of topographic maps (Thivierge and Marcus, 2007). Furthermore, we identified the influence of randomized firing activity (here modeled as external Poisson noise inputs to neurons) in this process. With moderate ACh modulation, noisy firing activity was able to disrupt and/or reverse potentiation patterns between network regions. In all, our results highlight the possible importance of localized spatio-temporal dynamics of ACh signaling in network reorganization and hence in memory formation.

## 2. Materials and methods

### 2.1. Cortical neuron model

We used a Hodgkin-Huxley based model of cholinergic modulation in pyramidal cells to simulate neuron membrane potential dynamics (Stiefel et al., 2009; Fink et al., 2013). It’s been shown that ACh signaling through M1 muscarinic ACh receptors can be well modeled by parameterizing the maximal conductance $g_{Ks}$ of a slow, low-threshold K^+^ mediated adaptation current. The model included a fast, inward Na^+^ current, a delayed rectifier K^+^ current and a leakage current as well. With C=1μF / cm^2^, units of V_i_ being millivolts and units of t being milliseconds, the current balance equation for the $i^{th}$ cell was:


$$\begin{array}{l} C\frac{dV_{i}}{dt}=-g_{Na}m_{i,\infty }^{3}h_{i}\left(V_{i}-V_{Na}\right)-g_{K_{dr}}n_{i}^{4}\left(V_{i}-V_{K}\right)\\ \;-g_{K_{s}}^{i}z_{i}\left(V_{i}-V_{K}\right)\;-g_{L}\left(V_{i}-V_{L}\right)+I_{\mathrm{drive}}^{i}-I_{syn}^{i}+I_{\mathrm{noise}}^{i} \end{array}$$

where a constant current $I_{\mathrm{drive}}^{i}$ was externally applied. $I_{\mathrm{drive}}^{i}$ is an nonspecific depolarizing or hyperpolarizing current that a cell receives and it provides constant offset to the resting membrane potential (if $I_{\mathrm{drive}}^{i}$ is subthreshold). It can also generate a constant spiking regime (at different frequencies), independent of the synaptic input (if $I_{\mathrm{drive}}^{i}$ is super-threshold). Finally, it also changes the response of the cell to incoming synaptic input due to the reduced voltage difference between achieved membrane potential and the threshold. $I_{syn}^{i}$ represented the synaptic current received by the $i^{th}$ neuron and $I_{\mathrm{noise}}^{i}$ was external noisy input current pulses dictated by a Poisson process (Poisson Rate at 2.5, 5, and 10 Hz) with amplitude of $6\mu A/cm^{2}$ and duration 1 ms.

For Na^+^ channel, activation is instantaneous with steady state function $m_{i,\infty }\left(V_{i}\right)={\left\{1+\exp\left[\left(-V_{i}-30.0\right)/9.5\right]\right\}}^{-1}$ . The inactivation gating variable $h_{i}$ was described by:


$$\frac{dh_{i}}{dt}=\frac{h_{\infty }\left(V_{i}\right)-h_{i}}{\tau_{h}\left(V_{i}\right)}$$

where $h_{\infty }\left(V\right)={\left\{1+\exp\left[\left(V+53.0\right)/7.0\right]\right\}}^{-1}$ and $\tau_{h}\left(V\right)=0.37+2.78{\left\{1+\exp\left[\left(V+40.5\right)/6.0\right]\right\}}^{-1}$ .

The kinetics of delayed rectifier $K^{+}$ current was gated by $n_{i}$ , the dynamics of which was governed by:


$$\frac{dn_{i}}{dt}=\frac{n_{\infty }\left(V_{i}\right)-n_{i}}{\tau_{n}\left(V_{i}\right)}$$

where $n_{\infty }\left(V\right)=\{1+\exp{\left[\left(-V-30.0\right)/10.0\right]}^{-1}$ and $\tau_{n}\left(V\right)=0.37+1.85{\left\{1+\exp\left[\left(V+27.0\right)/15.0\right]\right\}}^{-1}$ .

**Figure 1:**
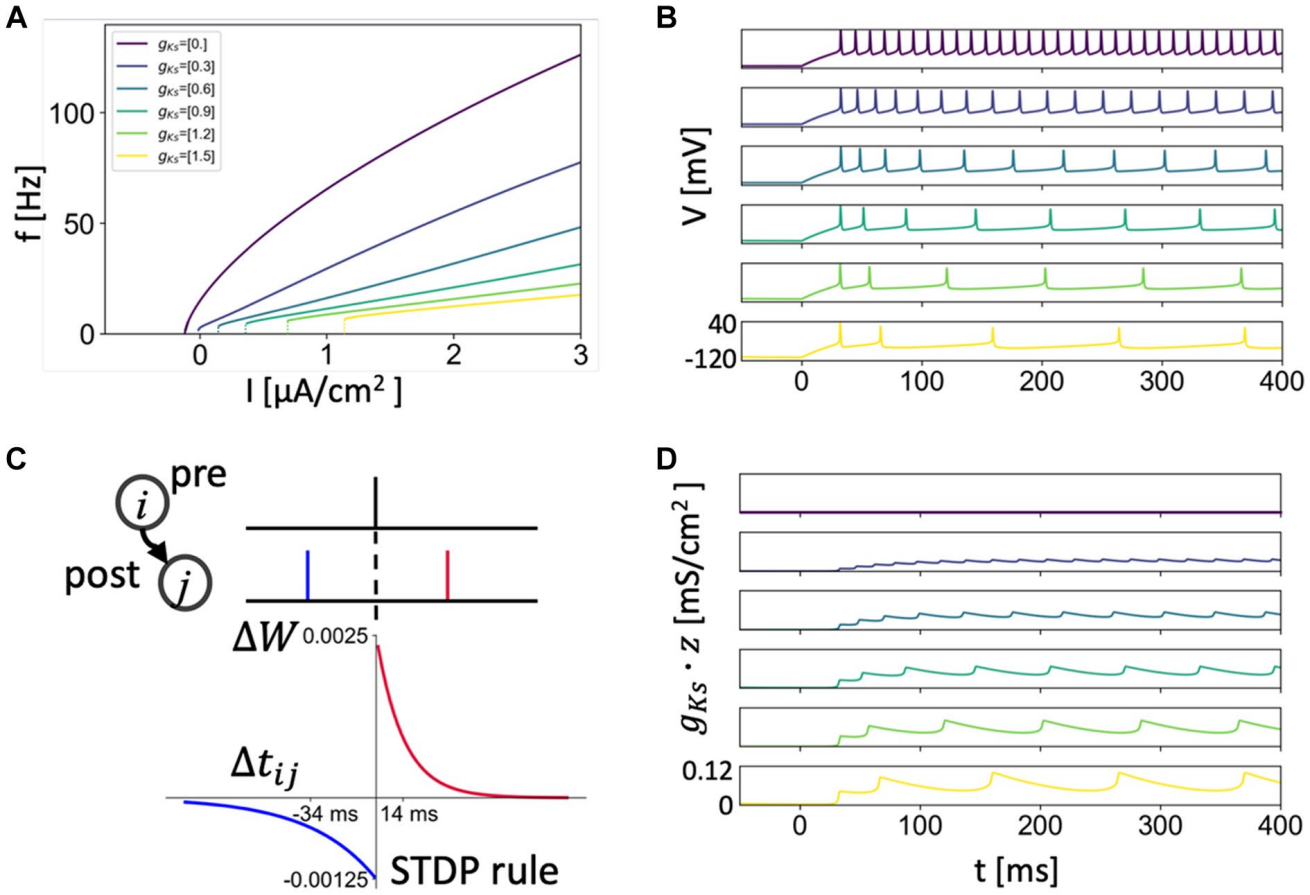
Frequency-current relationship, SFA and STDP rule. **(A)** Frequency-current curve of the neuron model with different *g_Ks_* values simulating different levels of ACh signaling (*g_Ks_* = 0 mS/cm^2^ for high ACh signaling and *g_Ks_* = 1.5 mS/cm^2^ for no ACh signaling). **(B,D)** Spike-Frequency-Adaptation (SFA) showed by the voltage traces **(B)** of different *g_Ks_* values with current of 1.5 μA/cm^2^ being applied (Same color code with **A** and other *g_Ks_* maps). SFA is caused by the slow build-up of the M-type K+ current during initial spiking as shown by its increasing conductance values g_Ks_* z **(D)**. **(C)** The asymmetric STDP rule. The potentiation decays with a time constant of 14 ms and depression decays with a time constant of 34 ms. For a given positive spike pair, the maximal amplitude for modification is 0.0025. For a given negative spike pair, the maximal amplitude for depression is 0.00125.

The gating variable $z_{i}$ of the slow, low threshold K^+^ current was governed by:


$$\frac{dz_{i}}{dt}=\frac{z_{\infty }\left(V_{i}\right)-z_{i}}{75.0}$$

where $z_{\infty }\left(V\right)={\left\{1+\exp\left[\left(-V-39.0\right)/5.0\right]\right\}}^{-1}$ . Values of other parameters were: $g_{Na}=24.0mS/cm^{2}$ , $g_{K_{dr}}=3.0mS/cm^{2}$ , $g_{L}=0.02mS/cm^{2}$ , $V_{Na}=55.0\mathrm{mV}$ , $V_{K}=-90.0\mathrm{mV}$ and $V_{L}=-60.0\mathrm{mV}$ . We varied the maximum conductance of the slow, low-threshold $K^{+}$ current in the $i^{th}$ cell, $g_{Ks}^{i}$ between 1.5 mS/cm^2^ for no ACh modulation and 0 mS/cm^2^ for strong ACh modulation. In this model neuron, decreasing values of $g_{Ks}$ increase membrane excitability as reflected in the frequency-current relation (Figure 1), as well as affect spike-frequency adaptation and the neural phase response curve (Fink et al., 2013; Roach et al., 2019).

### 2.2. Spike timing dependent potentiation (STDP) rule

We used an asymmetric STDP rule: the potentiation profile decayed with a time constant $\tau_{+}$ of 14 ms as a function of spike timing difference and the depression profile decayed with a time constant $\tau_{-}$ of 34 ms. For a positive spike timing difference (post-synaptic - pre-synaptic spike time), the maximal amplitude for modification $A_{+}$ was 0.0025. For a given negative spike timing difference, the maximal amplitude for depression $A_{-}$ was 0.00125 (Song et al., 2000). The STDP rule was implemented by adjusting the synaptic weight $w_{i,j}$ in time between presynaptic neuron i and postsynaptic neuron j by the following equation where $△t_{i,j}$ is the spike time difference between two cells (Figure 1C).

$$△w_{i,j}=\{\begin{array}{c} A_{+}e^{-△t_{i,j}/\tau_{+}}\;\mathrm{if}△t_{i,j}\geq 0\\ -A_{-}e^{△t_{i,j}/\tau_{-}}\;\mathrm{if}△t_{i,j}<0 \end{array}$$

Synaptic conductance values were constrained to remain in the interval between 0 and 0.01 mS/cm^2^.

### 2.3. Multi-module network model

**Figure 2:**
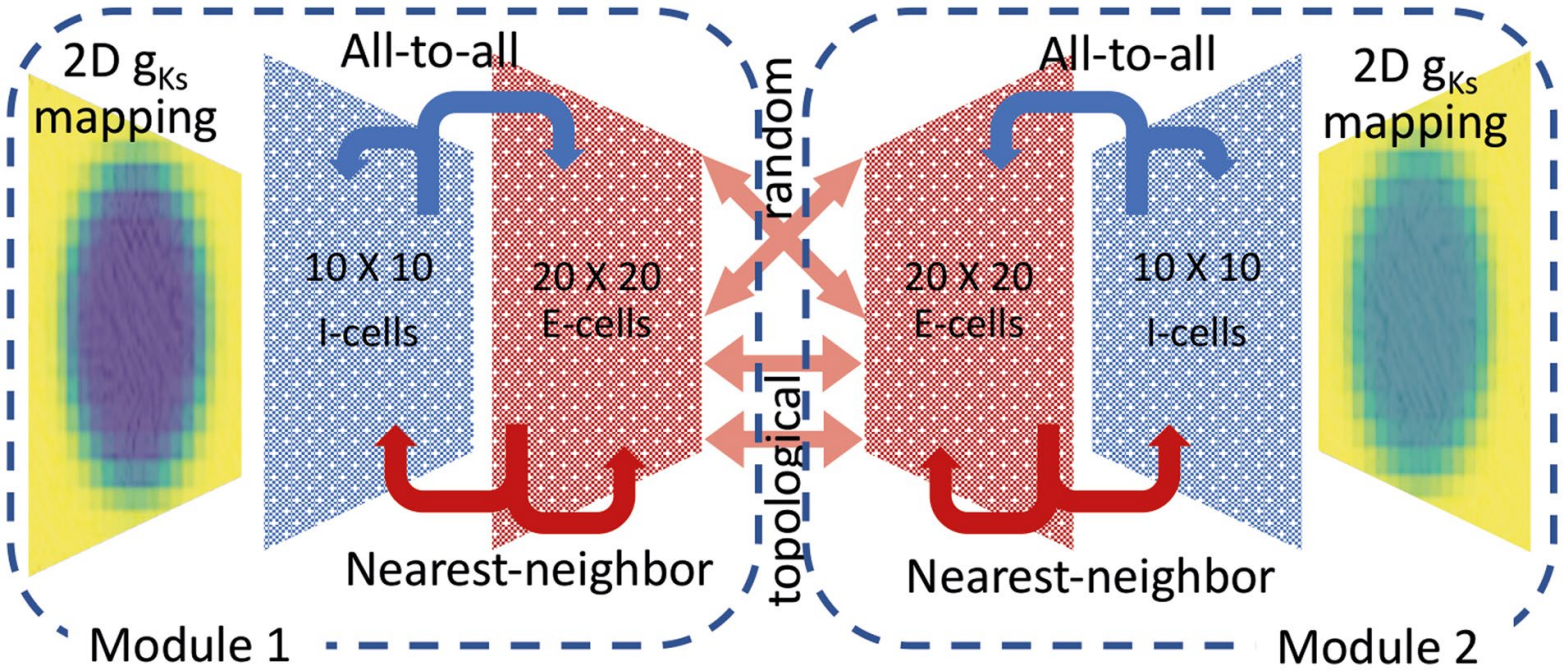
Schematic showing multi-module E-I network connectivity and spatial heterogeneous *g_Ks_* distribution. Two network modules are interconnected with plastic excitatory synaptic connections, that change via a STDP rule (see above) and connect randomly or topographically. Each module consists of two-dimensional networks with 400 excitatory (E) neurons and 100 inhibitory (I) neurons with local excitatory connectivity and global inhibitory connectivity. Within each module, the external input current to all neurons is homogeneous. Each module has its own spatially heterogenous *g_Ks_* mapping. Poisson noise is at the same frequency for both modules.

Model networks consisted of two modules synaptically coupled by excitatory synapses (Figure 2). Each module consisted of a two-dimensional (i.e., the neurons within the module are positioned on a plane, with connections distances calculated accordingly, see below) E-I network with the same network topology but varying *g_Ks_* values and DC input. We applied the STDP rule to only the inter-module connections between the E cells. The inter-module connectivity was one of two types: random or topographical. For randomly connected modules, each E-cell received 40 randomly selected incoming synapses from the other module. While when topographical connectivity was applied, each E-cell was connected to 40 nearest E-cells from the other module as if the two networks were placed on top of each other. The synaptic strength was set initially to 0.005 mS/cm^2^. The in-degree number was reduced to 5 in some simulations as noted in figure captions to better illustrate the potentiation patterns, as high local connectivity obliterated topological specificity (i.e., many neurons were receiving input from their firing neighbors and from the other module).

**Figure 3:**
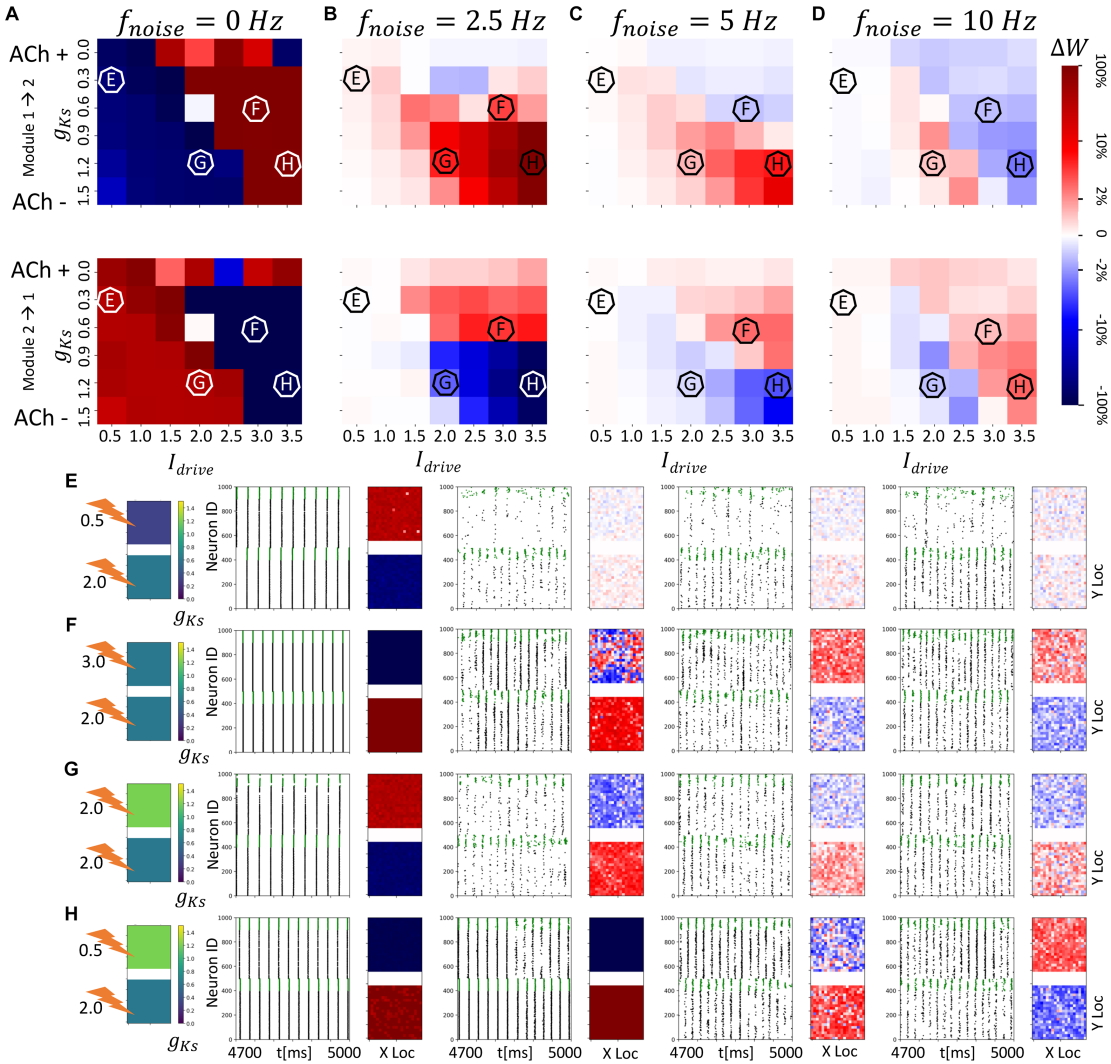
Noise disrupts and/or reverses the plasticity pattern between two randomly connected network modules with spatially homogenous *g_Ks_* distribution. The *g_Ks_* level and DC input for the module 2 are fixed at *g_Ks_* = 0.6 mS/cm^2^ and DC = 2.0 μA/cm^2^, respectively, while the corresponding parameters for the module 1 are varied as shown in the colormaps **(A–D)**. The noisy current inputs (modeled with a Poisson process) are applied at different frequency levels (0, 2.5, 5, 10 Hz – from left to right columns corresponding to **(A–D)**. The average change of synaptic weights ∆*w* is color coded with a linear-logarithmic scale shown on the rightmost colorbar (red: potentiation; blue: depression). **(A–D)** Average change in maximum synaptic conductance of connections: incoming to module 2 (top subplots); incoming to module 1 (bottom subplots). **(E–H)** correspond to example parameter values marked as E,F,G,H in **(A–D)**. In panel **(E–H)** rows, the leftmost plot shows the *g_Ks_* values for each network module (top: module 1; bottom: module 2; lighting symbol: I*_drive_*). The subsequent subplots (left to right) correspond to results with different frequencies of noise applied to both modules. Each example consists of 300 ms raster plot (left subplot) and a visualization of the change in weight for incoming synapses to each cell in the module (right subplot, red = potentiation, blue = depression). **(E)** The *g_Ks_* level and DC input for module 1 are 0.3 mS/cm^2^ and 0.5 μA/cm^2^. **(F)** The *g_Ks_* level and DC input for module 1 are 0.6 mS/cm^2^ and 3.0 μA/cm^2^, respectively. **(G)** The *g_Ks_* level and DC input for module 1 are 1.2 mS/cm^2^ and 2.0 μA/cm^2^, respectively. **(H)** The *g_Ks_* level and DC input for module 1 are 1.2 mS/cm^2^ and 3.5 μA/cm^2^, respectively.

Within each module, we used 400 excitatory (E) neurons and 100 inhibitory (I) neurons evenly distributed over separate square lattices ( 20×20 E cell lattice and 10×10 I cell lattice, Figure 2). The inhibitory cells accounted for 20% of cells similar to what has been reported experimentally in the cortex (Sahara et al., 2012). A local excitation-global inhibition network topology (like center-surround or lateral inhibition topologies) was used in which E cells sent outgoing connections to their 40 nearest neighbors on the E cell lattice and to their 10 nearest neighbors on the I cell lattice, (i.e., cells having the smallest distance, $r=\sqrt{i^{2}+j^{2}}$ , where i, j are neuron’s location indices; when needed, for the subset of cells that had the same distance r, we selected the adequate number of cells at random). Inhibitory cells sent outgoing connections to all E cells and all I cells. Periodic boundary conditions were imposed on cells near the lattice edges.

Local excitation and longer range inhibition, often referred to as “Mexican hat” organization, has been used in network models of orientation selectivity (Ben-Yishai et al., 1995; Douglas et al., 1995; Somers et al., 1995; Koch and Segev, 1998; Ernst et al., 2001), working memory in frontal cortex (Wang, 2001), and multiplicative neural responses in parietal cortex (Salinas and Abbott, 1996). Moreover, it was found that, in visual cortex, the long axons of GABAergic basket cells may provide the substrate for long-range inhibition (Buzás et al., 2001; Kang et al., 2003).

To illustrate network dynamics on a raster plot, we indexed neurons by lattice column such that a neuron’s index, $ID_{i}$ , was set to the sum of its lattice y-coordinate and the product of its lattice x-coordinate with the length of the lattice network, $ID_{i}=y_{i}+x_{i}\times L$ ( L=20 for E-cells and L=10 for I-cells). The first 400 indices were assigned to E-cells in the module 2 while the module 2 I-cells’ indices ranged from 401 to 500; the module 1 cells were similarly indexed as 501–1,000.

The synaptic current $I_{syn}^{i}$ represented the total synaptic current received by neuron i and was given by $I_{syn}^{i}=\underset{j}{\sum }I_{syn}^{ij}$ where $I_{syn}^{ij}=w_{ij}\underset{k}{\sum }\exp\left(-\frac{t-t_{jk}}{\tau }\right)\left(V_{i}-E_{syn}^{j}\right)$ at times $t>t_{jk}$ (spike time of $j^{th}$ neuron’s $k^{th}$ spike). The synaptic strength $w_{ij}$ is the $ij^{th}$ element in the adjacency matrix for the weighted directed graph for synaptic connections in our network model. For within module connections, we used 0.01 mS/cm^2^, 0.05 mS/cm^2^, 0.04 mS/cm^2^ and 0.04 mS/cm^2^ for E–E, E-I, I-I and I-E synaptic strengths, respectively. For all synaptic currents we used the same decay time constant τ=3.0ms . The reversal potential of the synaptic current ( $E_{syn}^{j}$ ) was set to 0 mV for excitatory synapses and −75 mV for inhibitory synapses.

### 2.4. Generation of heterogenous ACh spatial maps

To simulate spatially heterogeneous distribution of ACh levels, we constructed a mapping of maximal conductance values $g_{Ks}$ across the E cell and I cell lattices (Figure 2). The $g_{Ks}^{i,j}$ values for E cells, based on their i,j position in the 20×20 lattice (for E-cells) or 20×20 lattice (for I-cells) were given by


$$g_{Ks}^{i,j}=\min\left(g_{Ks}\right)+S\left(d_{i,j}-r\right)$$

where *S*(x) = 1/(1 + exp(−x)) is the standard sigmoid function, $d_{i,j}$ is the Euclidian distance to the nearest center of an ACh signaling hotspot, and r is the radius of the ACh signaling hotspot.

### 2.5. Measurements of network dynamics and potentiation patterns

All results presented here are averages over four trials, with each trial simulation having duration 5,000 ms and starting from random initial conditions. Network bursting frequency was calculated with summed voltage traces of E-cells in each module. To compute network level activation patterns, the discrete spiking times of every neuron were convolved with a Gaussian function (*σ* = 1 ms) centered at spike times. The convolved activation times of all neurons were then summed to form cumulative network traces. By finding peaks of these network activity traces, the bursting times can be identified for computation of mean phase coherence (MPC) (Mormann et al., 2000) between two modules:


$$MPC_{\mathrm{top},\mathrm{bottom}}=\frac{\sum_{k=1}^{N}e^{i\phi_{k}}}{N}$$

where $\phi_{k}=2\pi \frac{t_{\mathrm{bottom},k}-t_{\mathrm{top},k}}{t_{\mathrm{top},k+1}-t_{\mathrm{top},k}}$ in which $t_{\mathrm{top},k}$ is k^th^ bursting time of module 1 and $t_{\mathrm{bottom},k}$ is k^th^ bursting time of module 2. Similarly, we can compute $MPC_{\mathrm{bottom},\mathrm{top}}$ and take the average of two MPC values for the final MPC records.

The potentiation pattern was visualized based on a synaptic weight change matrix $\Delta W=100\%\times \left(W_{f}-W_{i}\right)/W_{i}$ , where $W_{i}$ is the adjacency matrix for intermodule connections representing initial synaptic weights and $W_{f}$ corresponds to the final adjacency matrix after 5-s simulations. We averaged ΔW across rows to compute the average maximal conductance change of incoming intermodule synaptic weights for each E-cell to visualize the pattern of potentiation. To compute the average change of weights across all incoming synapses to a given module, we averaged ΔW across all E-cells’ of that module. In addition, to better visualize synaptic weight changes, we used a linear-logarithmic scale in both the positive and negative directions from 0. Specifically, between −2 and 2% we used a linear scale while between −100% to −2 and 2 to 100% we used a logarithmic scale. This allows us to compare small and large changes in weight.

## 3. Results

In this study, we use biophysical excitatory-inhibitory (E-I) multi-module neural network models to elucidate how spatially heterogenous ACh signaling coupled with external stimuli can mediate spatially constrained potentiation patterns. While ACh acts through multifaceted mechanisms on neuronal and network level functions, we concentrate here on its influence on the K^+^ M-current (modulated through M1 receptors) and its consequences for network reorganization. With network connectivity within each module fixed in a local excitation/global inhibition topology, we focus on analyzing synaptic reorganization patterns, driven by STDP, of excitatory-to-excitatory synapses between the two network modules. We consider two types of inter-module network connectivity: random (the plastic synapses between E-cells are randomly organized with a fixed in-degree) and topographical (with the two modules aligned one on top of the other, E-cells in one module are connected to the nearest E-cells in the other module).

### 3.1. Muscarinic-mediated cholinergic modulation of neural response properties affect network dynamics

ACh modulation of the M-current exerts continuous control of neuronal excitability properties, leading to changes in network firing dynamics (Stiefel et al., 2009; Fink et al., 2013; Roach et al., 2019; Yang et al., 2021). We first review these cholinergic-induced changes that can impact network potentiation patterns under STDP. The K^+^ ion channels influenced by muscarinic receptor activation, and their corresponding ionic current, are blocked when ACh is high (Stiefel et al., 2009). We simulate these specific effects of ACh by decreasing the value of the maximal conductance of the K^+^ M-current, $g_{Ks}$ , such that low values of $g_{Ks}$ correspond to high ACh tone and high values of $g_{Ks}$ correspond to low ACh tone (Figures 1A,B,D).

Through this regulation of the M-current, ACh changes neural response to input such that for high and low levels of ACh modulation the neuron excitability changes between two archetypes: Type 1 and Type 2, respectively. These two excitability types differ in the dynamical mechanism of spike generation (Stiefel et al., 2008) which leads to several differences in input response characteristics between the two types, including a change in frequency and spike timings response to different current inputs. In terms of spike frequency, response to an injected current (f/I or gain function) (Tsuno et al., 2013), both excitability types (Type 1 and Type 2) have a critical current, $I_{c}$ , below which no spiking occurs, but are quite different in terms of spiking response around this point. Type 1 (high ACh) neurons will fire at arbitrarily small frequencies as the critical value of $I_{c}$ is reached leading to a continuous frequency-current curve, whereas Type 2 (low ACh) neurons have a discontinuous frequency increase from quiescence and initiate firing at a higher frequency (Figure 1A). Another critical feature difference between Type 1 and Type 2 excitability is that Type 2 neurons vary much less their firing rate in response to changes in injected current [i.e., have reduced gain (Tsuno et al., 2013)]. The difference in gain between these neuron types leads to increased firing responses to input for networks of Type 1 (high ACh) cells and larger differences in firing rates between cells receiving different inputs in Type 1 networks compared to Type 2 (low ACh) networks.

A concurrent change in response characteristics that occurs with ACh modulation of the M-current is differential response to brief, weak stimuli in terms of spike timing perturbation (i.e., advance or delay). This cellular property is quantified by the phase response curve (PRC) and can affect network synchronization propensity (Gutkin and Ermentrout, 1998; Gutkin et al., 2003; Izhikevich, 2005; Stiefel et al., 2008; Börgers, 2017). However, in our networks its effects may be reduced due to being in a strong coupling regime.

The cellular-level differences between high and low ACh modulation influence network firing dynamics and consequently plasticity patterns by STDP. Specifically, robust potentiation (and depotentiation) by STDP relies on consistent relative spike times between pairs of cells. Low ACh modulation may support such consistent firing as the majority of cells will fire within a network synchronous volley (also referred to it as a burst) whose duration is within the STDP time window. On the other hand, increased gain under high ACh modulation can cause feedback reverberatory firing between reciprocally connected cells whereby higher gain network regions may fire both before and after low gain network regions within the STDP time window. This can lead to different plasticity patterns between connected network regions. We thus show that a seemingly simple STDP rule can lead to diametrically different results that are modulated in part by ACh levels.

### 3.2. Differential neural excitability and external noise control patterns of synaptic rewiring in multi-module networks with random inter-module connectivity

In our networks, neural excitability is modulated by both *g_Ks_* level (ACh modulation) and direct current (DC) input. To understand the interplay between these two parameters in controlling network excitability and mediating differential synaptic reorganization patterns in our two-module networks, we varied both parameters *g_Ks_* and DC in module 1, while the parameters in network module 2 were held fixed. Additionally, both modules received external noisy input current pulses at different frequency levels.

We first consider spatially homogeneous ACh modulation in both modules with module 2 parameters set to *g_Ks_* = 0.6 mS/cm^2^ and DC = 2.0 μA/cm^2^, and these parameters varied in module 1. Within each module, the network exhibits synchronous volleys, with neurons within the modules being tightly synchronized, regardless of ACh level. This is due to the pyramidal-interneuron gamma (PING) mechanism (Börgers et al., 2005) produced by the local excitation – global inhibition connectivity, which we have previously linked to ACh modulated M-currents (Lu et al., 2020; Yang et al., 2021) and was shown in experimental studies (Howe et al., 2017). Given the small size of our model networks, we generally consider them to be subsets of synaptically coupled cells that are embedded within a larger brain-size network. As such, we do not consider that the synchronous firing observed in our results represents the firing activity observed from all neurons in a brain region. Instead our results represent firing of a subset of directly connected cells within a brain region.

With the STDP rule implemented on the excitatory synapses between modules, we observed that, depending on the relative excitability of the two modules, synaptic weights between modules are potentiated or depressed (Figure 3). Specifically, in the absence of noise input (Figure 3A), synaptic weights from the more excitable module to the less excitable module are potentiated ( ΔW positive). This can be expected since the more excitable module typically releases a highly synchronized spike volley first with the volley recruiting all cells in the module, and driving a similarly global volley in the less excitable module. In this regime, the modules may be viewed as acting like a coupled oscillator system in which the oscillator with higher natural frequency leads the oscillator with lower natural frequency.

**Figure 4:**
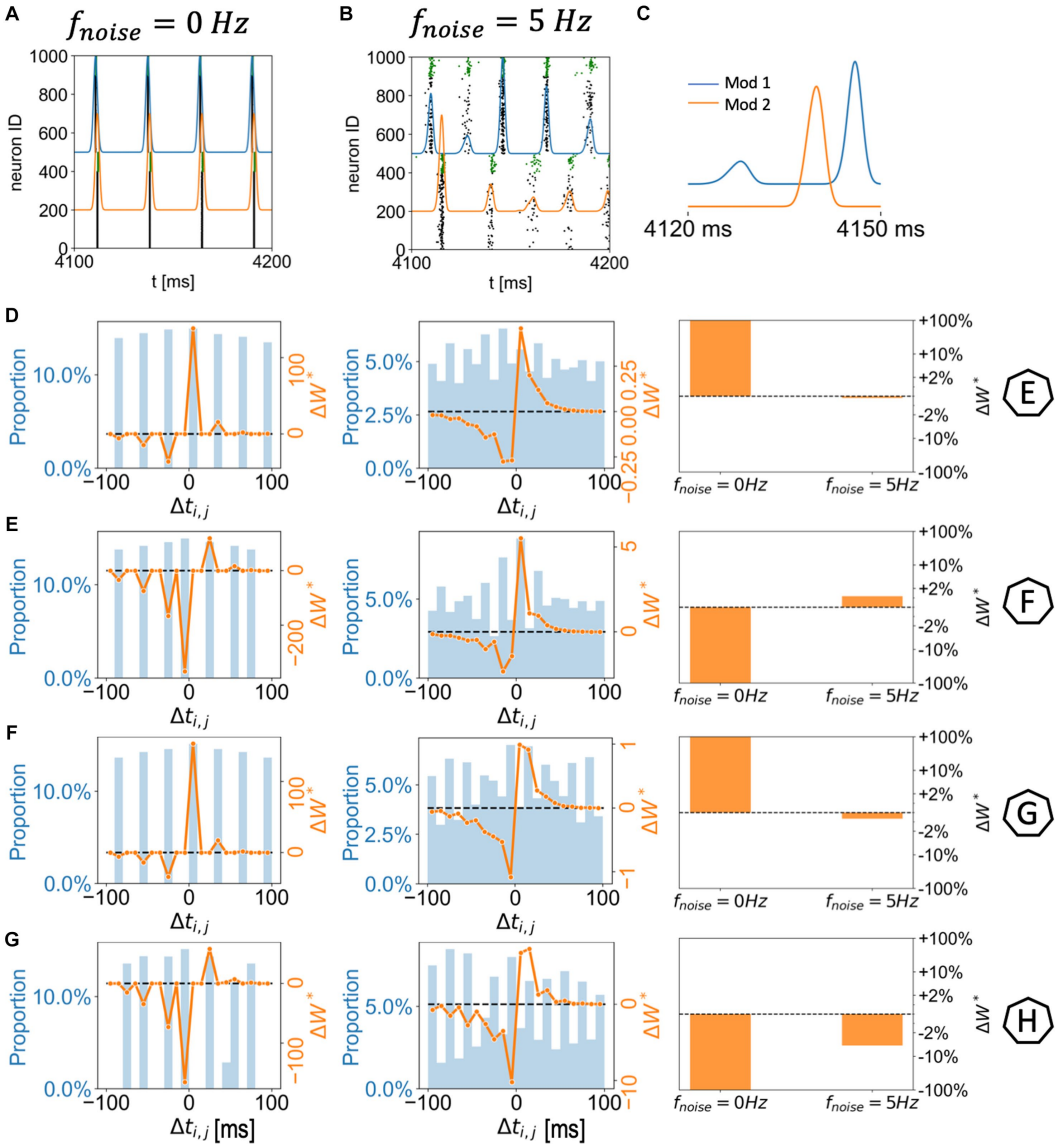
Network module bursting dynamics and synaptic potentiation in two randomly connected network modules with spatially homogenous *g_Ks_* distribution. **(A,B)** 300 ms raster plots with module network bursting traces (blue: module 1; orange: module 2). The *g_Ks_* level and DC input for module 1 are 0.6 mS/cm^2^ and 3.0 μA/cm^2^, respectively, (Example F in Figure 3). The *g_Ks_* level and DC input for module 2 are fixed at *g_Ks_* = 0.6 mS/cm^2^ and DC = 2.0 μA/cm^2^, respectively. The noisy current inputs are applied at different frequency levels (**A**: 0 Hz; **B**: 5 Hz). **(C)** Magnification of the module network bursting traces in the presence of noise **(B)**: the noise on the more excitable module (module 1) drives a small activity burst that in turn triggers a full spike volley in module 2; the module 2 volley triggers a secondary volley in module 1. **(D–G)** Correspond to example parameter values marked as E,F,G,H in Figure 3. The subplots (left to right) correspond to two (left: 0 Hz; middle: 5 Hz) histogram plots of spike pair time differences (between module 2 and module 1; positive spike pair difference means the E-cell in module 1 spikes first) during 4–5 s (left axes; blue) combined with overall synaptic potentiation curve weighted by STDP function (right axes; orange); And a summary bar plot of the overall synaptic potentiation under different noisy frequency levels.

As the *g_Ks_* value and DC input are varied in module 1, its excitability relative to module 2 changes as does the spike volley order between modules. This creates a diagonal boundary in the ΔW color maps of Figure 3A between parameter regions of potentiation and depotentiation of inter-module synaptic connections. On the left side of the diagonal boundary, module 2 is more excitable and thus, spike volleys in module 2 lead those in module 1 and this results in potentiation of synapses from module 2 to module 1 and depression of connections from module 1 to module 2. Detailed examples of these plasticity patterns are marked as E and G in Figure 3A. Conversely, on the right side of the diagonal boundary, module 1 is more excitable and hence spike volleys in module 2 follow those of module 1 and the plasticity pattern is reversed (examples on Figure 3A are marked as F and H).

However, when noise is applied to cells in both modules, these plasticity patterns are disrupted or even reversed as a function of noise frequency (Figures 3B–D). Specifically, as the frequency of applied noise increases (2.5, 5, and 10 Hz – Figures 3B–D, respectively), the change of the synaptic weights scales down significantly, and, critically, the synaptic reorganization pattern gradually switches in terms of which connections are potentiated or depressed. In particular, now synaptic weights from the less excitable module to the more excitable module are on average potentiated. As will be shown more clearly below, this is due to the fact that the synchronized spike volleys become more complex. Specifically, as the noise initiates the volley first in the more excitable module, that in turns triggers the volley in the less excitable module, that subsequently recruits cells in the more excitable module for a secondary volley. These volleys resemble more typically observed bursts of activity where an individual cell may fire multiple spikes over a longer time period. Here ACh plays a key role. Lack of ACh results in more hyperpolarization after a spike, due to activation of the m-current, thus effectively depressing the possibility of a secondary volley for the cells with reduced ACh levels. Conversely, high ACh concentration blocks the m-current, leading to higher sensitivity of the cell to the synaptic input and consequently promotion of a secondary volley.

We more closely analyzed a few examples of such changes in the plasticity pattern (marked on the color maps in Figures 3B–D as E, F, G and H, and further analyzed in Figure 4) to investigate how the reversal depends on the relative excitability of the two modules (see Supplementary Figure 1 for a comparison of network activity when STDP is absent). In Example E excitability of module 1 is significantly lower than for module 2 (in this case *g_Ks_* is lower in module 1, but it remains less excitable than module 2 because its DC input is lower; *g_Ks_* = 0.3; DC = 0.5). When there is no noise present (Figure 3E leftmost panels, 0 Hz noise frequency), bursting in the more excitable module (i.e., module 2) leads bursting in module 1 causing potentiation of synapses from module 2 to module 1 (red in module 1 incoming synaptic change plot) while synapses from module 1 to module 2 were depressed (Figure 4D left panel). Presence of noise of increasing frequency progressively disrupts this firing order with no clear potentiation pattern forming at any noise frequency (Figure 3E left to right panels and Figure 4D middle to right panels).

**Figure 5:**
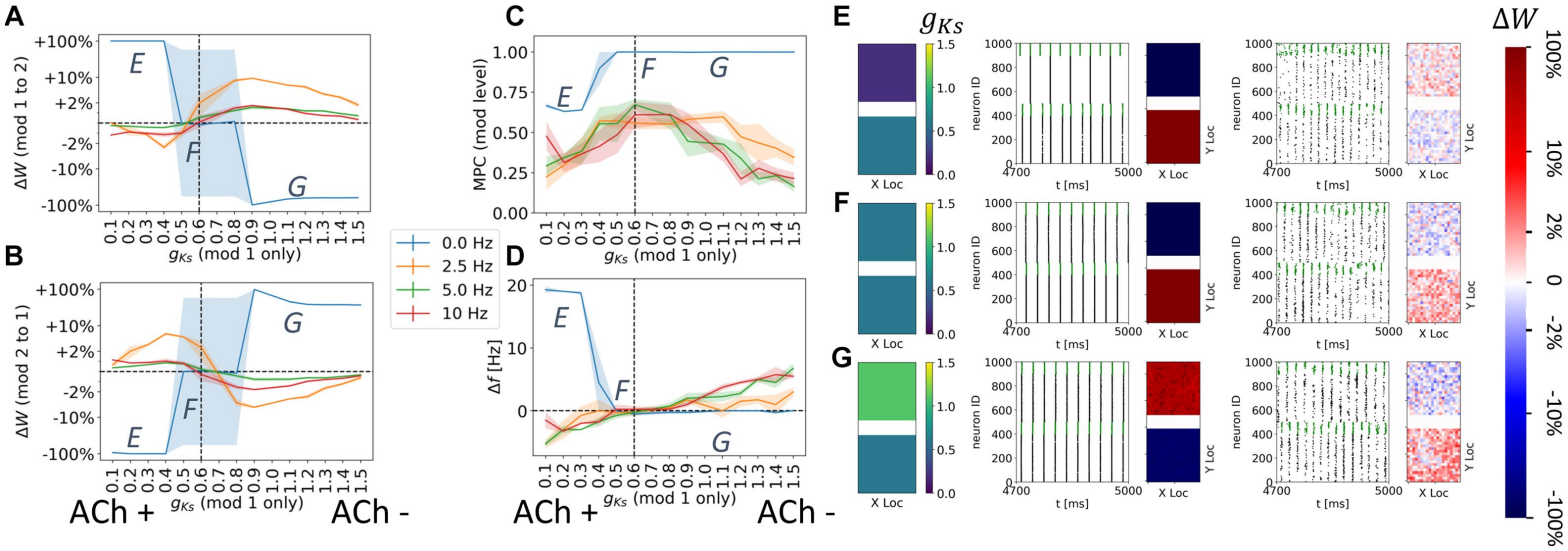
Noise reverses direction of synaptic potentiation when ACh levels are mismatched in the coupled modules. The DC input for both modules is fixed at DC = 2.0 μA/cm^2^. The *g_Ks_* value of the module 2 was set to be *g_Ks_* = 0.6 mS/cm^2^ (vertical dashed lines in **A–D**) while the *g_Ks_* value of the module 1 were uniformly varied to simulate different levels of ACh signaling. The noise (modeled as Poisson process) was simulated to be at different frequency (0, 2.5, 5, 10 Hz in legend). The average change of synaptic weights ∆*w* is color coded with a linear-logarithmic scale shown on the rightmost colorbar (red: potentiation; blue: depression). **(A)** Average change in maximum synaptic conductance of connections incoming to module 2 as a function of *g_Ks_* levels of module 1. **(B)** Average change in maximum synaptic conductance of connections incoming to module 1 as a function of *g_Ks_* levels of module 1. **(C)** Mean phase coherence between two modules’ synchronous volleys as a function of *g_Ks_* of the module 1. **(D)** Difference in bursting frequency between module 2 and module 1 as a function of *g_Ks_* of the module 1. **(E–G)** Examples marked as E,F,G in **(A–D)**. In panel **(E–G)** rows, the leftmost plot shows the *g_Ks_* values for each E-I network module (top: module 1; bottom: module 2). The next subplots correspond to different frequencies of noise applied to both modules. Each example consists of raster plot from 4,700 ms to 5,000 ms (left subplot) and a visualization of the change in weight for incoming synapses to each cell in the module (right subplot, red = potentiation, blue = depression). **(E)** The *g_Ks_* level and DC input for module 1 are 0.2 mS/cm^2^ and 2.0 μA/cm^2^. **(F)** The *g_Ks_* level and DC input for module 1 are 0.6 mS/cm^2^ and 2.0 μA/cm^2^. **(G)** The *g_Ks_* level and DC input for module 1 are 1.1 mS/cm^2^ and 2.0 μA/cm^2^.

On the other hand, Example F (Figures 3F, 4A–C,E) shows a reversal of the plasticity pattern with increasing noise frequency. In this case, with no noise added, module 1 is more excitable due to higher DC (*g_Ks_* values are the same in both modules) and its synchronous volleys systematically lead those of module 2. This causes strong synaptic potentiation from module 1 to module 2 (leftmost panel, 0 Hz, Figure 3F and Figure 4E left panel). With progressively higher noise frequency, the plasticity pattern switches as the spike volleys in module 1 become fragmented due to noise (i.e., only a limited subpopulation of neurons spike on the burst initiation) and, subsequently, synaptic feedback from module 2 spike volleys drive secondary spike volleys in module 1 reversing the order of the synaptic events. Specifically, as neurons in the more excitable module (module 1 on Figure 4C) are released from inhibition (earlier than those in module 2) and their voltage approaches threshold, the random noise triggers a small avalanche of activity within that module (Figure 4C). This burst in turn drives a synchronous volley in module 2, which finally triggers a secondary volley in module 1 (Figure 4C). The interaction between the spike volley of the less excitable module and the secondary spike volley in the more excitatory module causes reversal of the potentiation direction as compared to the no noise case. At a noise frequency of 2.5 Hz, bidirectional mean synaptic potentiation between the modules occurs, and the potentiation pattern is completely reversed at 5 Hz noise frequency and above (Figure 4E right panel). This is again mediated in part by varying ACh levels – a lack of ACh, causes hyperpolarization, via the m-current, after the spike thus effectively depressing the possibility of a secondary burst for the cells positioned in the regions with reduced ACh levels.

Similarly, in Example G (Figures 3G, 4F), module 2 is again somewhat more excitable than module 1, but now due to its higher *g_Ks_* value (the DC level is the same for both modules). Here, even at low noise frequency, there is a full reversal of the plasticity pattern obtained, as compared to no noise case. The potentiation reversal for lower noise frequencies is due to the closer match of excitability levels of both modules, together with higher *g_Ks_* of module 1 promoting more robust synchronization in module 1 and emergence of feedback secondary bursts in module 2, and reversal of synaptic events (Figure 4F).

Finally, example H (Figures 3H, 4G) illustrates the scenario when the switch of the plasticity pattern only happens at high frequency noise. Even though module 1 has high *g_Ks_*, it is more excitable because of its high DC level. Low noise has little effect on synchronization with high *g_Ks_* in module 1 thus the firing pattern is preserved. When there is no noise (leftmost panels, 0 Hz), module 1 leads module 2 in all rounds of bursts. Synapses from module 1 to module 2 are potentiated (red in module 2, Figures 3H, 4G) while synapses from module 2 to module 1 were depressed. Only for highest noise frequency (10 Hz) is this firing dynamic disrupted and the plasticity pattern reverses.

### 3.3. ACh alone may mediate reversal of synaptic potentiation

While the above results summarize the effects of combined changes in neural excitability due to varying *g_Ks_* and DC input, the excitability changes due to only varying *g_Ks_* can generate the reversal in synaptic plasticity pattern in the presence of noisy inputs. To show this, we investigated rewiring patterns between the modules having spatially homogenous but different *g_Ks_* values, the same value of DC current input, and random inter-module connectivity.

We observed that away from the balanced excitability state, synapses from the more excitable module to the less excitable module are potentiated when noise is absent (Figures 5A,B; compare to Figure 3A). Similarly to what we showed earlier, the presence of noise not only disrupts but also reverses this synaptic potentiation direction (Figures 5A,B; compare to Figures 3B–D). Specifically, when the ACh concentration in module 1 is high (low *g_Ks_* levels), incoming synapses to module 1 are potentiated when noise is present and depressed when the noise is absent. On the other hand, when the concentration of ACh in module 1 is lower (higher *g_Ks_* levels), incoming synapses to module 1 are depressed in presence of noise and potentiated in the absence of noise. The magnitude of synaptic changes is inversely proportional to the noise frequency.

**Figure 6:**
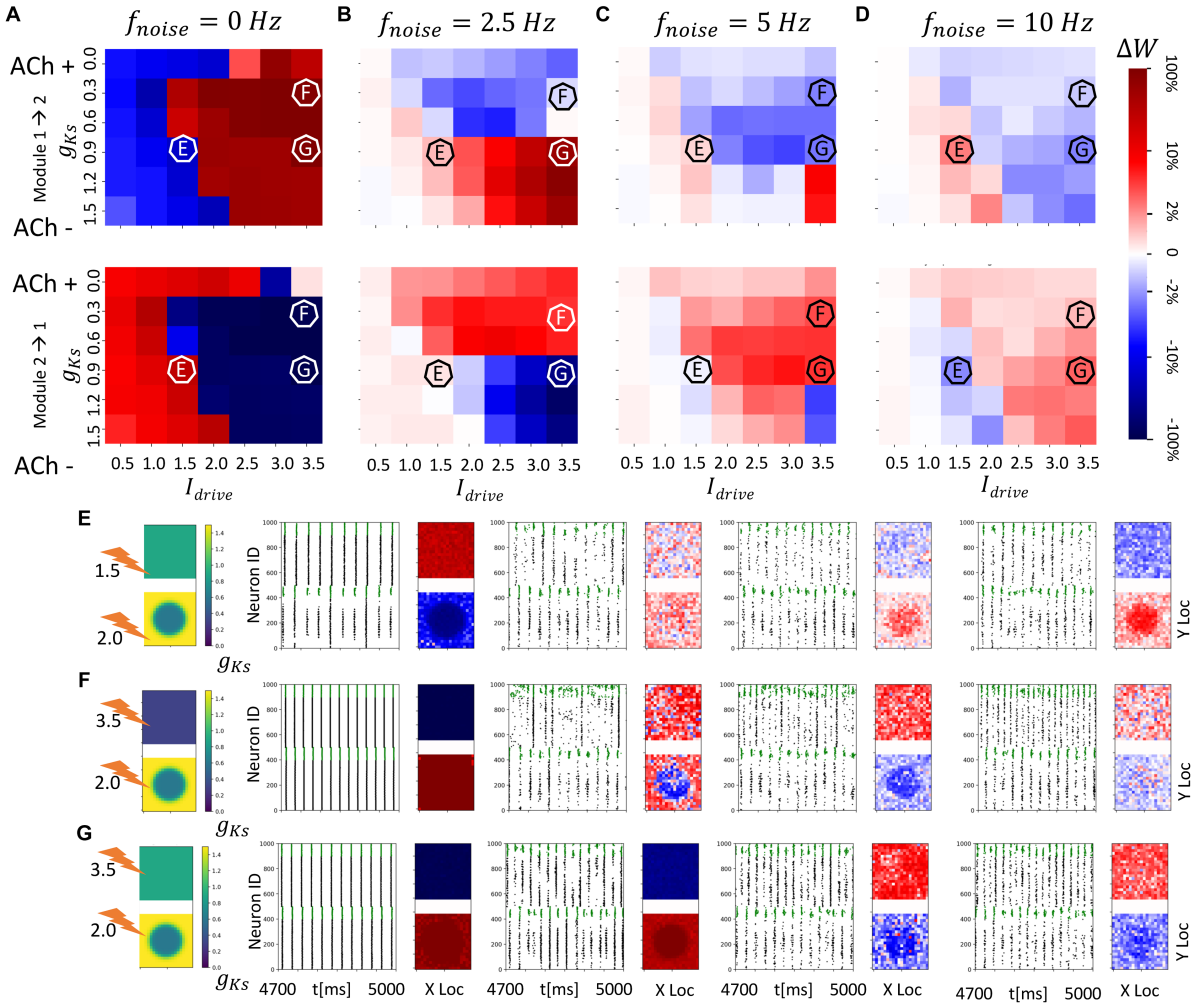
Spatial localization of plasticity induced by spatially heterogenous ACh modulation in two randomly connected network modules (module 2 has a *g_Ks_* hotspot and module 1 has homogenous *g_Ks_* distribution). The *g_Ks_* level and DC input for module 2 are fixed at *g_Ks_* = 0.6 mS/cm^2^ and DC = 2.0 μA/cm^2^, respectively, for the hotspot and *g_Ks_* = 1.5 mS/cm^2^ and DC = 3.0 μA/cm^2^, respectively, for the surroundings while the corresponding parameters for module 1 are varied as shown in the colormap. The noise (modeled with poisson process) was simulated to be at different frequency (0, 2.5, 5, 10 Hz from left to right columns corresponding to **A–D**). The change of synaptic weights is all color coded with logarithmic scale shown on the rightmost colorbar (red: potentiation; blue: depression). **(A–D)** Average change in maximum synaptic conductance of connections incoming to module 2 from module 1 (top subplot); to module 1 from module 2 (bottom subplot). **(E–G)** Examples marked as E,F,G in **(A–D)**. *g_Ks_* level and DC input for module 2 are fixed at 0.6 mS/cm^2^ and 2.0 μA/cm^2^, respectively, while the corresponding parameters for module 1 are varied as shown in the colormap. The noise (modeled with poisson process) was simulated to be at different frequency (0, 2.5, 5, 10 Hz from left to right columns). In panel **(E–G)** rows, the leftmost plot shows the *g_Ks_* values for each E-I network module (top: module 1; bottom: module 2; lighting symbol: I*_drive_*). The next subplots correspond to different frequencies of noise applied to both modules. Each example consists of raster plot from 4,700 ms to 5,000 ms (left subplot) and a visualization of the change in weight for incoming synapses to each cell in the module (right subplot, red = potentiation, blue = depression). **(E)** The *g_Ks_* level and DC input for module 1 are 0.9 mS/cm^2^ and 1.5 μA/cm^2^. **(F)** The *g_Ks_* level and DC input for module 1 are 0.3 mS/cm^2^ and 3.5 μA/cm^2^, respectively. **(G)** The *g_Ks_* level and DC input for module 1 are 0.9 mS/cm^2^ and 3.5 μA/cm^2^, respectively.

In terms of burst dynamics, tight phase locking of bursts in the two modules is only observed in the networks without noise (Figure 5C). In this case, burst locking is observed when the excitability of the two modules is matched (*g_Ks_* = 0.6 in both modules) and remains tight as module 1 becomes more excitable with higher values of *g_Ks_* (Figure 5C). When noise is present, on the other hand, phase locking between the modules was significantly lower, with peak locking when the modules have matched excitability (*g_Ks_* = 0.6), but lower locking when excitability is mismatched.

When no noise is present and when excitability of the two modules is similar (*g_Ks_* = 0.5 ~ 0.8 in the module 1), large variance in the plasticity pattern is observed across multiple runs because of varying initial conditions and simulated network connectivity that may additionally change relative module excitability (Figures 5A,B). This is due to the network bursting dynamics remaining unchanged during the simulation, which is further promoted by unidirectional strengthening of inter-module synapses, resulting in a potentiation pattern that is biased toward one direction based on initial conditions (example from one run shown in Figure 5F).

The reversal effect is driven by two characteristics of the ACh blocked m-current. Blockage of the m-current (high ACh, low *g_Ks_*) increases module excitability causing the noise to trigger a small, partial volley on that module, that consequently triggers the cascade as described above. At the same time, the module having low ACh (high *g_Ks_*) experiences the brunt of the slow hyperpolarizing m-current just after firing its volley, effectively stopping it from firing a secondary volley later, after the secondary volley of the more excitable module.

### 3.4. Spatial localization of synaptic reorganization due to ACh spatial heterogeneity of a single hotspot in multi-module networks with random inter-module connectivity

To further investigate how spatially heterogeneous ACh distributions mediate network reorganization patterns between randomly interconnected modules, we analyze directionality of synaptic potentiation when module 2 has a spatially heterogenous *g_Ks_* distribution in the shape of a single hotspot or bump. Specifically, within the hotspot *g_Ks_* and DC values are set to *g_Ks_* = 0.6 mS/cm^2^ and DC = 2.0 μA/cm^2^, respectively, while the surrounding portions of the network have lower excitability (*g_Ks_* = 1.5 mS/cm^2^ and DC = 3.0 μA/cm^2^). In module 1, the *g_Ks_* distribution is spatially uniform and we again vary *g_Ks_* values and DC input levels. Here the m-current, via *g_Ks_*, is the major factor driving high excitability within the hotspot even though the DC outside the hotspot is higher.

Results for this scenario basically mirror those discussed above where the excitability of module 1 compared to the excitability within the *g_Ks_* hotspot in module 2 dictates the plasticity pattern and that pattern may be reversed with the addition of noise (Figures 5A–D). Importantly, however, even though the modules have randomly assigned interconnections the plasticity pattern can exhibit spatial localization. For example, when the hotspot in module 2 is more excitable than module 1 and noise is absent, bursts in the hotspot, with sporadic recruitment of all neurons in module 2, lead the network synchronous spike volley in module 1 (Figure 6E). This dynamical pattern results in strong depression of the synapses incoming to the hotspot, and weaker depression of incoming synapses to neurons outside the hot spot. Incoming synapses to module 1, on the other hand, are uniformly potentiated. When noise is added, the synaptic reorganization pattern reverses while retaining spatial localization, with incoming synapses to neurons in the hotspot potentiating to a higher degree than in the surrounding neurons.

**Figure 7:**
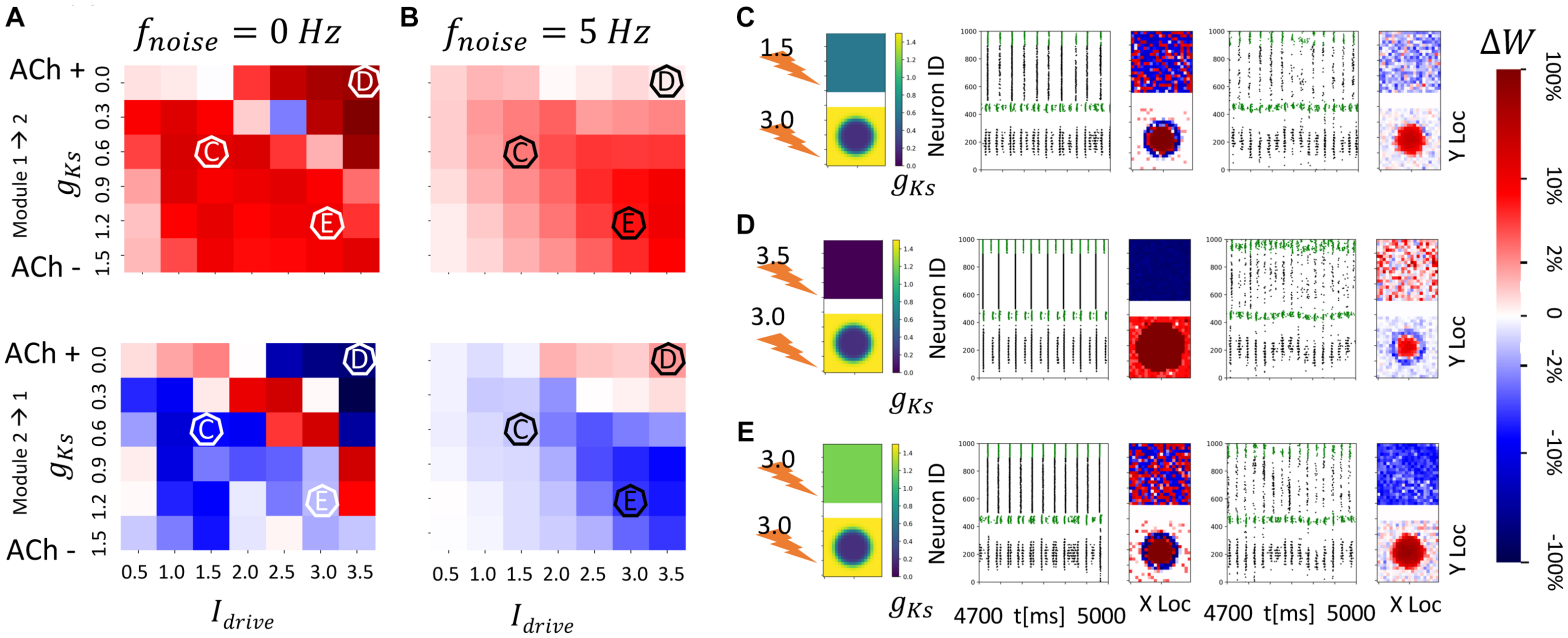
Consistent and spatially localized plasticity patterns for high and spatially heterogeneous *g_Ks_* (ACh) modulation between two randomly connected network modules. The *g_Ks_* level and DC input for module 2 are fixed at *g_Ks_* = 0.2 mS/cm^2^ and DC = 3.0 μA/cm^2^, respectively, for the hotspot and *g_Ks_* = 1.5 mS/cm^2^ and DC = 3.0 μA/cm^2^, respectively, for the surroundings while the corresponding parameters for module 1 are varied as shown in the colormap. The noise (modeled with Poisson process) was simulated to be at different frequency (0, 5 Hz from left to right columns corresponding to **A,B**). The change of synaptic weights is all color coded with logarithmic scale shown on the rightmost colorbar (red: potentiation; blue: depression). **(A,B)** Average change in maximum synaptic conductance of connections incoming to module 2 from module 1 (top subplot); to module 1 from module 2 (bottom subplot). **(C–E)** Examples marked as C,D,E in **(A,B)**. In panel **(C–E)** rows, the leftmost plot shows the *g_Ks_* values for each E-I network module (top: module 1; bottom: module 2; lighting symbol: I*_drive_*). The next subplots correspond to different frequencies of noise applied to both modules. Each example consists of raster plot from 4,700 ms to 5,000 ms (left subplot) and a visualization of the change in weight for incoming synapses to each cell in the module (right subplot, red = potentiation, blue = depression). **(C)** The *g_Ks_* level and DC input for module 1 are 0.6 mS/cm^2^ and 1.5 μA/cm^2^. **(D)** The *g_Ks_* level and DC input for module 1 are 0.0 mS/cm^2^ and 3.5 μA/cm^2^. **(E)** The *g_Ks_* level and DC input for module 1 are 1.2 mS/cm^2^ and 3.0 μA/cm^2^.

When the excitability of module 1 is significantly higher than the excitability within the ACh hotspot in module 2 (Figure 6F), for the no noise case, incoming synapses to module 2 are uniformly strengthened, due to bursting in module 1 leading module 2 volleys. Interestingly, for low noise frequency, the plasticity pattern is only partially reversed. As expected, incoming connections to module 1 become uniformly potentiated, but in module 2, only connections incoming to the hotspot are depotentiated. Connections targeting the neurons surrounding the hotspot remain potentiated. This is due to the fact that module 1 fires secondary volleys in response to synchronous volleys in module 2 due to its high excitability and these volleys occur after the cells in the hotspot fire but before the sparse activation of the cells surrounding the hotspot. For larger noise frequencies, this nonuniform plasticity pattern disappears and complete reversal of the plasticity pattern occurs with all synapses incoming to module 1 getting potentiated and the ones targeting module 2 being depotentiated irrespective of whether the targeted neurons are in the ACh hotspot or not.

As a final example, when lower *g_Ks_* and high DC in module 1 promotes volley synchronization, reversal of the plasticity patterns occurs only for larger noise frequencies (Figure 6G). There is not pronounced spatial localization of the plasticity in module 2 since the leading synchronous spike volleys in module 1 drive firing across the whole network of module 2.

In contrast, when neurons in the ACh hotspot in module 2 are highly excitable (high DC = 3 μA/cm^2^ and low *g_Ks_* = 0.2 mS/cm^2^), a more consistent plasticity pattern occurs regardless of the excitability of module 1 or the noise frequency (Figure 6). Specifically, synapses incoming to module 2 are generally potentiated while synapses incoming to module 1 are mostly weakened (Figures 7A,B). This is due to the fact that the high excitability within the hotspot results in secondary volleys in module 2 that are driven by synchronous volleys in module 1, readily observed when the noise is absent (Figures 7C–E, left panels). The time difference of volleys between the modules is conducive to potentiating synapses incoming to the neurons within the hotspot in module 2. The neurons outside the hot spot fire more sparsely, because of the high hotspot firing and global inhibition within the module, leading to non-significant synaptic changes at these cells, and high spatial localization of plasticity.

**Figure 8:**
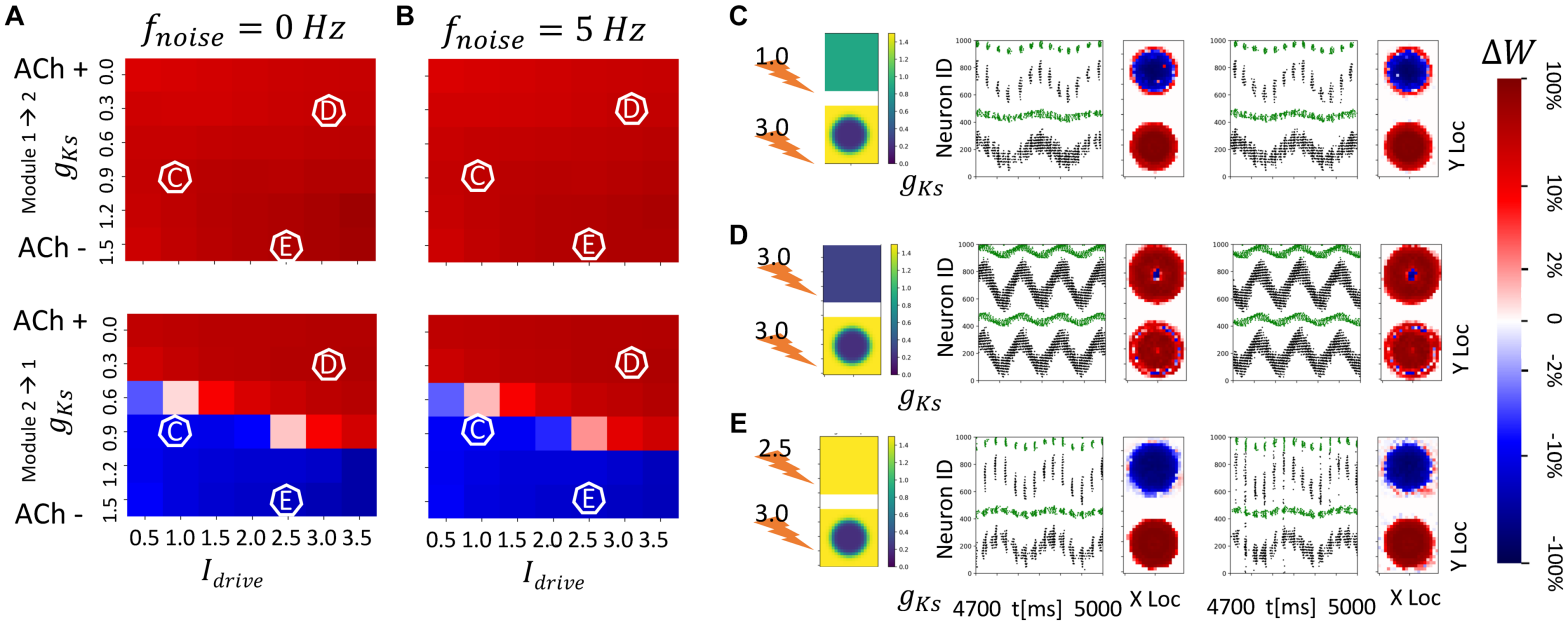
Strong spatial localization of plasticity with spatially heterogeneous ACh modulation between two topographically (nearest neighbor) connected network modules (module 2 has a strong *g_Ks_* hotspot and module 1 has homogenous *g_Ks_* distribution). The *g_Ks_* level and DC input for module 2 are fixed at 0.2 mS/cm^2^ and 3.0 μA/cm^2^, respectively, for the hotspot and 1.5 mS/cm^2^ and 3.0 μA/cm^2^, respectively, for the surroundings while the corresponding parameters for module 1 are varied as shown in the colormap. The synaptic connectivity in-degree is reduced to 5 in these networks (see Materials and methods). The noise (modeled with poisson process) was simulated to be at different frequency (0, 5 Hz from left to right columns corresponding to **A,B**). The change of synaptic weights is all color coded with logarithmic scale shown on the rightmost colorbar (red: potentiation; blue: depression). **(A,B)** Average change in maximum synaptic conductance of connections incoming to module 2 from module 1 (top subplot); to module 1 from module 2 (bottom subplot). **(C–E)** Examples marked as C,D,E in **(A,B)**. In panel **(C–E)** rows, the leftmost plot shows the *g_Ks_* values for each E-I network module (top: module 1; bottom: module 2; lighting symbol: I*_drive_*). The next subplots correspond to different frequencies of noise applied to both modules. Each example consists of raster plot from 4,700 ms to 5,000 ms (left subplot) and a visualization of the change in weight for incoming synapses to each cell in the module (right subplot, red = potentiation, blue = depression). **(C)** The *g_Ks_* level and DC input for module 1 are 0.9 mS/cm^2^ and 1.0 μA/cm^2^. **(D)** The *g_Ks_* level and DC input for module 1 are 0.3 mS/cm^2^ and 3.0 μA/cm^2^. **(E)** The *g_Ks_* level and DC input for module 1 are 1.5 mS/cm^2^ and 2.5 μA/cm^2^.

Only when the excitability of module 1 is greater than that of the *g_Ks_* hotspot in module 2 is there a weak reversal of potentiation pattern in the presence of noise (Figure 7D). In this case, the synapses incoming to module 1 are generally potentiated when noise is present. This is again due to the timing of secondary volleys in module 1 driven by the activity generated in module 2.

### 3.5. Strong spatial localization of plasticity patterns is induced by spatially heterogeneous ACh modulation when network modules are topologically connected

We also investigated how the structural network reorganization proceeds when the inter-module connectivity was topographically arranged at the start of the simulation. We consider a single, highly excitable ACh hotspot in module 2 and spatially uniform *g_Ks_* in module 1 with varying values and DC levels. Because of the local excitation-global inhibition connectivity within the modules, high hotspot firing suppresses activity of the surrounding neurons in module 2, and the topographical inter-module connectivity drives similar activity patterns in module 1. Therefore, little synaptic reorganization is detected outside the hotspot region in module 2, or the region directly connected to the hotspot in module 1, and plasticity is strongly spatially localized.

In these networks, synapses targeting the hotspot region in module 2 are potentiated regardless of the excitability of module 1 or noise frequency (Figure 8). When module 1 is significantly less excitable than the *g_Ks_* hotspot, incoming synapses to module 1 are depressed regardless of the presence of noise (Figures 8C,E), but when module 1 has similar or higher excitability than the hotspot these synapses are potentiated (Figure 8D). This is due to the fact that the spatial locations of activity within the hotspot (module 2) and its corresponding region in module 1 are synchronized. When both regions are highly excitable (as in Figure 8D) the activity consists of prolonged, multispike, synchronized high frequency spike volleys. This, due to the higher firing rates and asymmetric nature of the STDP rule, leads to preferential reciprocal potentiation of the inter-module connections.

The lack of noise dependence is due to the fact that local connectivity provided via topological inter-module connectivity leads to highly heterogenous inputs to individual cells in the modules, with cells inside the hotspot and those connected to it firing at high frequency and all other cells remaining quiescent. This leads to temporally asynchronous cell activation within the volleys independent of noise presence.

### 3.6. Topographical connectivity between spatially constrained regions of ACh modulation promotes reciprocal localized synaptic potentiation

**Figure 9:**
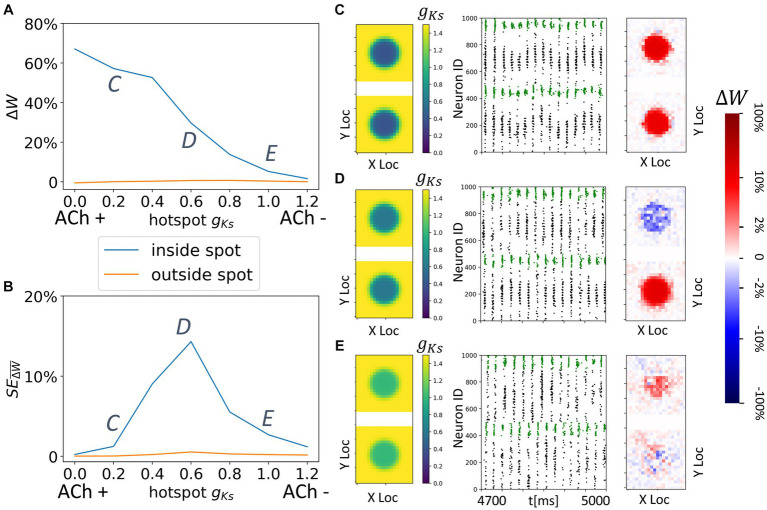
Spatially localized potentiation with spatially constrained *g_Ks_* modulation in both topographically connected network modules. **(A)** Incoming synaptic weight change percentage inside the hotspot (blue) and outside the hotspot (orange) as a function of the hotspot *g_Ks_* values. **(B)** Standard error across multiple trials of incoming synaptic weight change percentage inside the hotspot (blue) and outside the hotspot (orange) as a function of the hotspot *g_Ks_* values. **(C–E)** *g_Ks_* mapping of both modules visualization (left subplot; top: module 1; bottom: module 2), raster plot of 4.7 s to 5 s (middle) and a visualization of the change in weight for incoming synapses to each cell in the module (right subplot, red = potentiation, blue = depression). The average change of synaptic weights ∆*w* is color coded with a linear-logarithmic scale shown on the rightmost colorbar (red: potentiation; blue: depression). Parameters corresponding to C,D,E in **(A,B)** subplots.

Finally, we investigate the interaction of colocalized hotspots in both modules in networks having topological inter-module connectivity (Figure 9). As before module activation is predominantly limited to the hotspot locations. This leads to reorganization of only the synapses emanating from and/or targeting the two hotspots (Figure 9A). Here, we vary only *g_Ks_* values of the hotspots, keeping these values the same across the modules. We observe that when *g_Ks_* values are low, reciprocal connections targeting both hotspots are robustly potentiated (Figures 9A,B), forming strong, spatially localized intermodular connectivity between the hotspots (Figure 9C). This is due to synchronized multi-spike bursting in both hotspots (Figure 9C) and the asymmetric STDP rule. Interestingly, for intermediate values of *g_Ks_* the hotspots compete to drive synaptic potentiation (Figure 9D). Depending on the (random) initial conditions, the bursting pattern evolves randomly so that volleys generated by one of the hot spots lead those generated by the other one. As a result, synapses targeting one spot are potentiated whereas the reciprocal connections are weakened. This random direction of the plasticity pattern is evident in the high values of the standard deviation of synaptic changes (Figure 9B) for this range of *g_Ks_* values. Since the change can be positive (potentiation) or negative (depotentiation) the standard deviation of this randomized effect is large.

For higher values of *g_Ks_* within the hotspots, sparse synchronous spike volleys occur in the two modules that are not robustly synchronized (Figure 9E). This leads to an overall decrease in synaptic plasticity making it also less specific to the hotspots.

### 3.7. Spatially heterogeneous ACh modulation generates spatially constrained plasticity patterns that are influenced by the relative excitability of synaptically connected cells in topographically connected network modules

**Figure 10:**
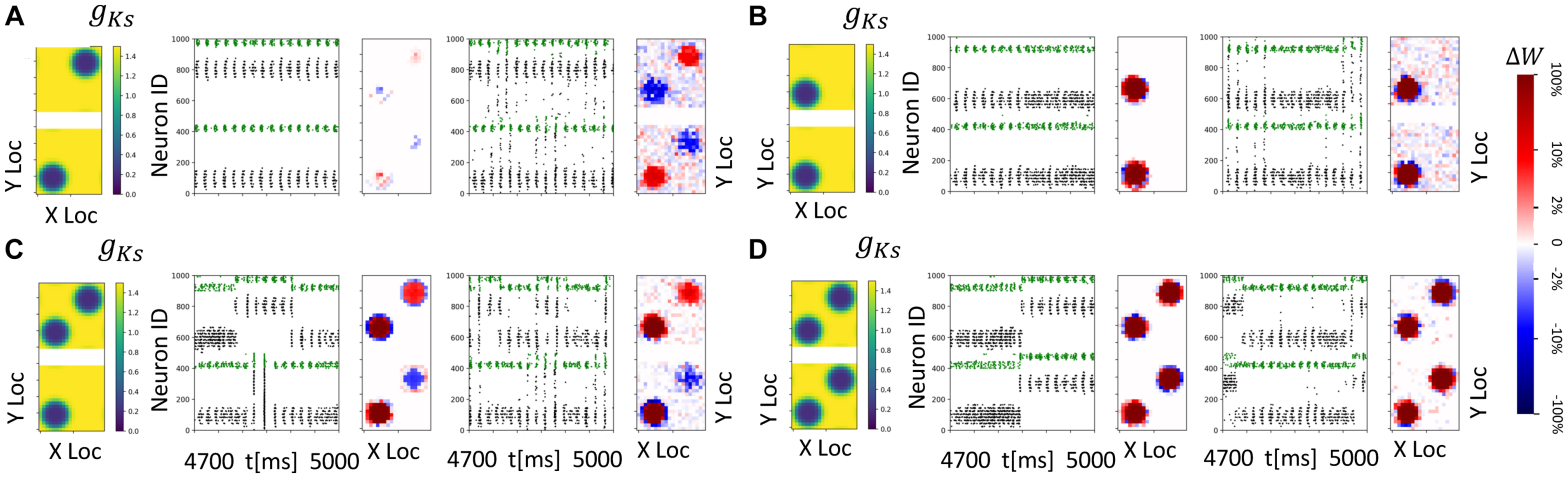
Spatially heterogeneous ACh modulation generates spatially constrained plasticity patterns in topographically connected network modules. For all subplots, the DC inpu ist 3.0 μA/ cm^2^ in both modules and *g_Ks_* values are 0.2 mS/cm^2^ at hotspots and 1.5 mS/cm^2^ in the background. **(A–D)** From left to right, panels show: *g_Ks_* mapping of both modules visualization (leftmost subplot; top: module 1; bottom: module 2), raster plot of 4.7 s to 5 s with no noisy inputs (2nd from left) illustrating a portion of E cell (cells 1–400 for module 2; cells 501–900 for module 1) and I cell (cells 401–500 for module 2; cells 901–1000 for module 1) firing patterns. The pixel color indicates cell type in each module (black: E-cells; green: I-cells). and a visualization of the change in weight for incoming synapses to each cell in the module with no noisy inputs (3rd subplot, red = potentiation, blue = depression). The average change of synaptic weights ∆*w* is color coded with a linear-logarithmic scale shown on the rightmost colorbar (red: potentiation; blue: depression); and spike raster plot (4th subplot) and incoming synaptic weight changes (5th subplot) with noisy inputs at 5 Hz.

Finally, we investigate patterns of network reorganization when both modules have multiple (up to two) strongly modulated (*g_Ks_* = 0.2 mS/cm^2^) cholinergic hotspots, in networks with topological inter-module connectivity. Cells outside the hotspots have low ACh modulation (*g_Ks_* = 1.5 mS/cm^2^) and, although DC input is high (3.0 μA/cm^2^ in both modules), the majority of firing activity occurs within the hotspots, even in the presence of noise. As observed in previous results (Yang et al., 2021), if a module contains two hotspots then firing activity switches over time between them generating theta band modulation of the gamma rhythm firing (Figures 10C,D).

Here the results are consistent with those obtained in previous sections. In general, (1) if the hotspots in the two modules share connections, these connections are strengthened reciprocally (Figures 10B–D); and (2) if the hotspots in the two modules do not have reciprocal connections, the connections incoming to the hotspot are potentiated, whereas connections outgoing from a hotspot to a non-modulated network region are weakened (Figures 10A,C). The presence of noisy inputs did not disrupt these plasticity patterns.

## 4. Discussion

Traditionally, cholinergic signaling has been assumed to be slow in terms of its concentration changes and with general spatial homogeneity. Recent evidence has shown, however, that ACh signaling is more spatially localized and asynchronous within activated brain modalities (Hasselmo, 1999; Dayan and Yu, 2002; Hasselmo and Giocomo, 2006; Sarter and Lustig, 2019). In our previous study of E-I networks with local excitatory connectivity and global inhibitory connectivity (Yang et al., 2021), we demonstrated that spatially heterogenous distributed ACh signaling can generate spatially localized gamma rhythms within high ACh modulated areas and, additionally, theta-gamma rhythmicity across spatially distinct ACh modulated areas. The coupled theta-gamma rhythmicity is regarded as a hallmark of attentive cognitive information processing (Colgin, 2015) in cortical and hippocampal areas, with experimental results showing that ACh modulation plays an important role in promoting this firing pattern (Newman et al., 2013; Howe et al., 2017). In this study, we built upon the previous modeling work and implemented ACh modulation in conjunction with spike timing dependent plasticity (STDP) on excitatory synapses between differentially modulated network regions in order to investigate the combined interactions of ACh-induced gamma rhythmicity and synaptic plasticity under the effects of spatially selective ACh modulation.

Using a multi-module network structure, we show that spatially heterogenous effects of cholinergic modulation via muscarinic M1 receptors can lead, when coupled with STDP, to reorganization of network structure, with noise (i.e., stochastic inputs) playing an important part in the process. Specifically, we show that synapses targeting localized regions with higher concentration of ACh (leading to lower activation of the K^+^ M-current) are selectively potentiated. This effect is primarily driven by increased cellular excitability which can also be induced by higher direct current (DC) input. Noise-induced firing affects the direction of synaptic potentiation between network regions leading to a preferred potentiation of synapses incoming to regions with higher excitability. When connected network regions are in similar excitability states, synaptic potentiation can be reciprocal if excitability is sufficiently high but otherwise potentiation is competitive with directional potentiation determined by specifics of firing activity. Our results suggest that ACh modulation can locally coordinate and govern directionality of synaptic potentiation between connected network areas and highlight the possible importance of spatial localization of ACh signaling in network reorganization and, hence, in feature binding and memory formation.

While STDP alters synaptic weights on the timescale of milliseconds, network reorganization, and subsequently learning, extends to a much slower timescale (Brzosko et al., 2019). The process of neuromodulation can be crucial in closing this gap. Cholinergic signaling has been shown to play an essential role in varied memory and learning processes. In particular, ACh modulation has been shown to have diverse and significant effects on synaptic plasticity and STDP (Seol et al., 2007; Picciotto et al., 2012; Brzosko et al., 2019; Fuenzalida et al., 2021). ACh has been shown to have priming effects on plasticity induction when it is present before the plasticity-inducing spiking activity, as well as affecting bias for potentiation or depotentiation when concurrently present (see Brzosko et al., 2019 for review). Results suggest these effects are mediated by multiple mechanisms, acting through both muscarinic (mAChR) and nicotinic (nAChR) receptor pathways. It has been shown that both mAChRs and nAChRs, localized pre- and post-synaptically, are crucial for synaptic plasticity in the hippocampus (Drever et al., 2011). However, cholinergic modulation of STDP has very complex effects. For example, muscarinic M1 receptor activation has been shown to enable induction of depotentiation (Seol et al., 2007; Brzosko et al., 2017) regardless of the timing sequence of pre- and post-synaptic spikes while, when both receptor subtypes are activated, it has been shown that potentiation has been facilitated regardless of the spike timing sequence (Sugisaki et al., 2016). Another study (Ovsepian et al., 2004) demonstrated that muscarinic receptor activation lowered the threshold for LTP induction and further results identified the postsynaptic M1 mAChR activation being crucial in the modulation of hippocampal synaptic plasticity (Shinoe et al., 2005). The diversity of reported effects of ACh on STDP indicate a highly complex dependence on the ACh concentration present, the cholinergic receptor subtypes that are activated, the cell types and brain region affected as well as the specific spike firing patterns that induce plasticity.

These complex cholinergic mechanisms may work in tandem to contribute to behavioral learning. It was shown that cholinergic regulation of learning-induced synaptic plasticity can be mediated through the activation mAChRs and imparts the contextual fear learning-driven strengthening of hippocampal excitatory pyramidal synapses through the synaptic incorporation of AMPA-type glutamate receptors (AMPARs) (Mitsushima et al., 2013). At the same time, contextual fear learning also enhances the strength of inhibitory synapses on hippocampal pyramidal CA1 neurons, in a manner mediated by the activation of nAChRs (Mitsushima et al., 2013).

Moreover, localized ACh release and subsequent localized network reorganization can critically underlie feature binding, with synapses targeting regions with high ACh being potentiated while those targeting other regions remaining weak or being actively depotentiated. Recent research suggests that ACh, and specifically the muscarinic receptor pathway, may be critical to feature binding. A recent study found that the muscarinic cholinergic antagonist scopolamine selectively impaired the ability of rats to learn a cross-modal odor-texture feature-conjunction (FC) task, but not their ability to learn a future-singleton task. In addition, scopolamine left the retrieval of previously learned FC stimuli intact (Botly and De Rosa, 2007). Similar results were observed in humans when their attention was disrupted (Botly and De Rosa, 2008).

Here we focus on a different mechanism for the influence of ACh on synaptic potentiation. Namely, that instead of directly modulating the cellular mechanisms underlying STDP, ACh affects synaptic potentiation by its local modulation of neuronal excitability and thus firing activity. Our modeling results show that modulation of M1 receptor activation can change neuronal excitability that, in turn, leads to synaptic potentiation of synapses targeting neurons in regions of upregulated ACh levels. This mechanism is specifically driven by increased firing response of the neurons that are located in high ACh regions, leading to reverberatory firing activity within the time window of STDP action. It possibly explains experimental results (Shinoe et al., 2005) showing that a low concentration (50 nm) of carbachol enhanced long-term potentiation (LTP) of excitatory synaptic transmission in mouse hippocampal slices. Significantly for our results, this enhancing effect was abolished in M_1_ mAChR knock-out mice but not in M_3_ mAChR knock-out mice, although LTP itself was intact in both mutant strains.

While robust potentiation (or depotentiation) through STDP requires consistent firing patterns between neurons, neural activity in the brain is highly variable with the variability possibly playing a critical role in brain function (Uddin, 2020). Our modeling results demonstrate that noise (i.e., stochastic inputs) can play an important role in the reorganization of network structure. Neuronal activity exhibits substantial irregularity, and STDP with complex timing within spike patterns has been well discussed (Caporale and Dan, 2008 and see below). We show that the presence of noise inputs generating more variable firing patterns significantly scales down the change in synaptic weights between two network modules. In addition, the synaptic reorganization pattern progressively switches the direction of potentiation between network regions as the variability of firing increases (Figures 3, 5, 6). On the other hand, strong ACh modulation can constrain the direction of potentiation regardless of DC input and noisy inputs (Figure 7).

In this study, STDP was implemented with a history-independent, spike pair-based, canonical Hebbian plasticity rule (Song et al., 2000). Since its discovery by Bi and Poo in hippocampal cultures (Bi and Poo, 1998), STDP has been observed in numerous types of synapses and highly complex effects have been identified on synaptic modification depending on the specific firing patterns of pre- and post-synaptic cells as well as the state of the post-synaptic neuron (Markram et al., 1997; Sjöström et al., 2001; Froemke et al., 2010; Feldman, 2012). For example, at hippocampal CA3-CA1 synapses, variations in the frequency and duration of pre- and post-synaptic firing patterns can alter plasticity from a canonical STDP rule to rules that favor only potentiation or depotentiation (Wittenberg and Wang, 2006). Similarly, at cortical synapses, higher frequency firing rates have been associated with the promotion of synaptic potentiation and lower firing rates with depotentiation, regardless of spike timing (Markram et al., 1997; Sjöström et al., 2001; Nelson et al., 2002; Zilberter et al., 2009). Additionally, in the visual cortex, synaptic modification has been shown to depend not just on the time interval between a pair of spikes but also on the timing of preceding spikes (Froemke and Dan, 2002; Froemke et al., 2006). Specifically, synaptic modification can be attenuated if the post-synaptic cell fires within a short time window before the occurrence of the plasticity-inducing pre-post spike pair, and when both pre- and post-synaptic cells fire multiple times within a short time window, other processes such as short-term depression, further affect synaptic modification. Taken together, the diversity of synaptic modifications observed under different conditions of firing rates, synaptic cooperativity and postsynaptic voltage suggest that spike timing on the millisecond time scale is only one component of the synaptic plasticity process and this component may play a major role in determining synaptic modification in some circumstances, and may be negligible in others (Feldman, 2012).

Different computational models for synaptic plasticity have been developed to try to account for these complex dependencies of spiking timing on synaptic modification. For example, models have been implemented that take into account the effect of multiple pre- and post-synaptic spike combinations [for example pre – post spike triplets or quadruplets, reviewed in Morrison and Gettelman (2008)]. Taking a more biophysical approach, models have been introduced in which synaptic modification depends on the dynamics of post-synaptic intracellular calcium concentration driven by cell spiking (Shouval et al., 2002; Scherz-Shouval et al., 2010). The study by Graupner et al. (2016) directly compared the synaptic plasticity patterns predicted by models of these different types to both artificial and *in vivo* recorded spiking activity. They found that all three types of rules (canonical STDP, multi-spike STDP and calcium-based) predicted similar responses at low firing rates but the multi-spike STDP and calcium-based plasticity rules better predicted the promotion of synaptic potentiation at high firing rates, as has been observed experimentally at some synapses.

Despite our use of a canonical, pair-based STDP plasticity rule, our results suggest that variation of cellular excitability (and thus firing rates), induced by ACh blockade of the K+ m-current, can generate variations in plasticity patterns and a promotion of symmetric synaptic potentiation at high excitability and firing rates. This effect is most pronounced in the results shown in Figures 7, 8, 9C, 10 where the ACh modulation is high in the *g_Ks_* hotspot in module 2 (Figures 7, 8) or in the *g_Ks_* hotspots in both modules (Figures 9A, 10). In these results, incoming synapses to firing cells in both modules potentiate when the firing cells are under high ACh modulation. For the results in Figures 3–6, the cellular excitability level in at least one of the modules is at a moderate level and asymmetric, bidirectional plasticity patterns more generally occur, consistent with previously cited findings at lower firing rates. Thus, our results suggest that cellular excitability can additionally contribute to the myriad ways that STDP may be modulated.

Overall, this in-silico study draws inspirations from recent advancements in understanding ACh signaling and proposes mechanisms for the effects of M1 mAChR activation on synaptic plasticity. Experimental evidence has overwhelmingly demonstrated the crucial role of ACh signaling in synaptic plasticity. Here, we show that cholinergic modulation can regulate network firing dynamics, interacting with external input as well as network topology, to impact synaptic reorganization via STDP. As it has been challenging to untangle specific effects of ACh signaling on synaptic plasticity, our modeling approach can provide a comprehensive understanding of certain mechanisms contributing to the effects of ACh signaling on synaptic plasticity. At the same time, understanding the mechanisms underlying cholinergic regulation of learning-induced synaptic plasticity may help inform experimental studies of learning and memory.

## Data availability statement

The datasets generated for this study can be found in the GitHub repository HeterACh-STDP: https://github.com/YihaoYang/HeterACh-STDP.

## Author contributions

VB and MZ conceived the research idea. YY performed all simulations and data analysis. YY, VB, and MZ wrote the manuscript. All authors contributed to the article and approved the submitted version.
