# Supplementary material for: Acetylcholine facilitates localized synaptic potentiation and location specific feature binding
Source: Front Neural Circuits. 2023 Nov 10;17:1239096. doi: 10.3389/fncir.2023.1239096 (PMC10684311; doi:10.3389/fncir.2023.1239096)
Supplement: Supplementary file 1 [file Image_1.pdf]

Acetylcholine facilitates localized synaptic potentiation and location specific feature binding

Yihao Yang[1], Victoria Booth[2*], Michal Zochowski[3*],

[1]Department of Physics, University of Michigan, Ann Arbor, MI, United States of America
[2]Departments of Mathematics and Anesthesiology, University of Michigan, Ann Arbor, MI, United States of America
[3]Department of Physics and Biophysics Program, University of Michigan, Ann Arbor, MI, United States of America

*Corresponding authors

E-mail: michalz@umich.edu (MZ) and vbooth@umich.edu (VB)

Supplemental Figure 1

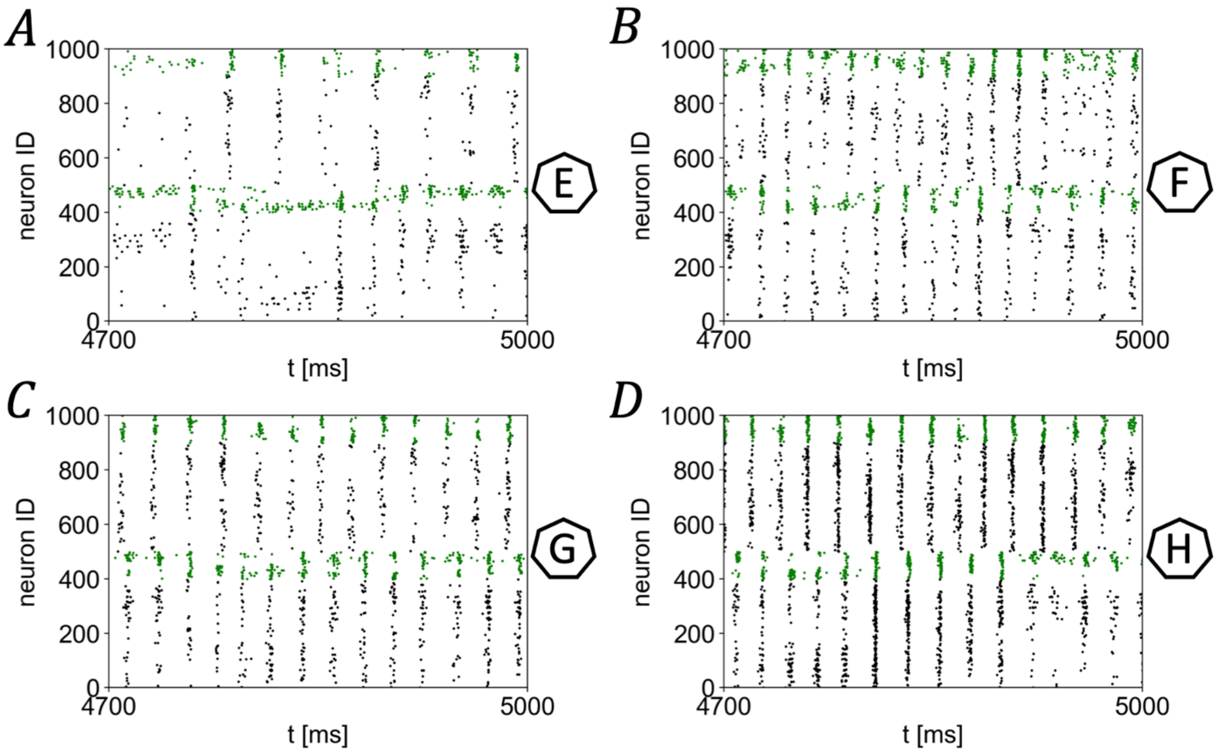

Supplementary Figure 1. **Network dynamics in the absence of plasticity.** (**A-D**) Raster plots (black: E-cells; green: I-cells) of 300 ms for the example cases E, F, G, H with 5 Hz noise as in the results shown in the 3rd column of Figure 3 E,F,G,H, except without STDP. The g_{Ks} level and DC input for module 2 are fixed at g_{Ks}=0.6 mS/cm^2 and DC=2.0 μA/cm^2, respectively. (**A**) The g_{Ks} level and DC input for module 1 are 0.3 mS/cm^2 and 0.5 μA/cm^2. (**B**) The g_{Ks} level and DC input for module 1 are 0.6 mS/cm^2 and 3.0 μA/cm^2 respectively. (**C**) The g_{Ks} level and DC input for module 1 are 1.2 mS/cm^2 and 2.0 μA/cm^2 respectively. (**D**) The g_{Ks} level and DC input for module 1 are 1.2 mS/cm^2 and 3.5 μA/cm^2 respectively.
